# In-hospital outcomes of adults with diabetes treated in certified vs. non-certified hospitals: a nationwide analysis of German DRG statistics

**DOI:** 10.3389/frhs.2026.1782882

**Published:** 2026-05-21

**Authors:** Marie Auzanneau, Andreas Fritsche, Alexander J. Eckert, Esther Seidel-Jacobs, Martin Heni, Stefanie Lanzinger

**Affiliations:** 1 Institute of Epidemiology and Medical Biometry, Ulm University, Ulm, Germany; 2 German Center for Diabetes Research (DZD), Neuherberg, Germany; 3 Department of Internal Medicine IV, University Hospital Tübingen, Tübingen, Germany; 4 Institute of Diabetes Research and Metabolic Diseases (IDM) of the Helmholtz Center Munich at the University of Tübingen, Tübingen, Germany; 5 Institute for Biometrics and Epidemiology, German Diabetes Center (DDZ), Leibniz Center for Diabetes Research at Heinrich-Heine-University Düsseldorf, Düsseldorf, Germany; 6 Department for Diagnostic Laboratory Medicine, Institute for Clinical Chemistry and Pathobiochemistry, University Hospital Tübingen, Tübingen, Germany; 7 Division of Endocrinology and Diabetology, Department of Internal Medicine 1, University Hospital Ulm, Ulm, Germany

**Keywords:** accreditation, certification, diabetes care, inpatient care, quality improvement

## Abstract

**Introduction:**

Certifications for inpatient diabetes care aim to improve quality of care and patient safety. However, the benefits of treatment in hospitals certified by the German Diabetes Association (DDG) have not yet been evaluated.

**Methods:**

In the national diagnosis-related groups (DRG) statistics for the 3-year period 2021–2023, we analyzed all inpatient cases with diabetes (all types, primary or secondary diagnosis) aged ≥20 years. Odds ratios for in-hospital mortality and differences in the length of hospital stay were compared between diabetes certified (DCH) and non-certified hospitals (NDCH) using hierarchical multiple regression models.

**Results:**

Of 43.4 million total inpatient cases in 2021–2023 in Germany, 8.1 million (18.5%) had a documented diabetes diagnosis. The prevalence of diabetes was 19.0% in the 300 DCH compared to 18.3% in the 1,103 NDCH, but 60.7% of all inpatients with diabetes were treated in NDCH. Overall, the mortality for inpatients with diabetes in DCH was lower (3.11% [95%-CI: 3.09–3.14] vs. 3.16% [3.14–3.18], OR: 0.98 [0.97–0.99], *p* = 0.002) and the hospital stay was shorter (7.93 days [7.79–8.07] vs. 8.96 days [8.87–9.05], *p* < 0.001) than in NDCH. After stratification, the differences in mortality in DCH vs. NDCH were significant for cases with diabetes as primary diagnosis (OR for type 1 diabetes: 0.59 [0.49–0.70], OR for type 2 diabetes: 0.87 [0.84–0.91], both *p* < 0.001), with ketoacidosis (DKA): 0.88 [0.80–0.97], *p* = 0.009), and with hypoglycemia: 0.79 [0.76–0.83], *p* < 0.001), but not for cases with diabetes as secondary diagnosis.

**Conclusion:**

This nationwide analysis indicates a significantly reduced mortality in diabetes certified hospitals for people with diabetes as primary diagnosis, with DKA or hypoglycemia. Prospective comparisons stratified by hospital characteristics are needed to deepen these results.

## 1 Introduction

Like in many other countries, diabetes is highly prevalent in Germany, with more than 9 million people living with diagnosed condition (1, 2), and at least 2 million more estimated to have undiagnosed diabetes (1). According to the International Diabetes Federation (IDF), Europe had the second highest number of diabetes-related deaths in 2021 (approximately 1.1 million) after the Western Pacific region (3).

Although diabetes is typically managed in an outpatient setting, one in five inpatients in Germany has a diabetes diagnosis (4). Moreover, up to 40% of inpatients in internal medicine, surgery and neurology departments of maximum care hospitals have diabetes (5), underscoring the importance of effective inpatient diabetes management.

Diabetes is associated with a high economic and health burden for patients and represents a major cost factor for the healthcare system (3, 6, 7). It is associated with need for surgery at a younger age, more frequent complications, prolonged hospital stays, and higher in-hospital mortality (4, 8). Depending on the forecast, an estimated 11 million people will be living with diabetes in Germany by 2040 or 2050 at the latest (2, 9), posing serious challenges for the healthcare system.

In several countries, like in Denmark (10), Belgium (11), Australia (12) or the USA (13), implementation of diabetes-specific hospital accreditations has led to improvements in the care of patients with diabetes. In the UK, the Diabetes Care Accreditation Programme (DCAP) was officially launched in 2023 to improve inpatient care and provide assurance that national standards for diabetes care in hospital are being met equally across hospitals (14). The assessment of pilot program has already indicated significant improvement in all of the pilot sites (14).

In Germany, the German Diabetes Association (DDG) confers certifications for hospitals that demonstrate evidence-based, guideline-compliant diabetes care and maintain appropriately qualified medical personnel (15). However, the potential benefits of these certifications for adults with diabetes compared to non-certified hospitals have not yet been systematically evaluated. In this analysis, we compared the mortality rates and the length of stays for adults with diabetes treated in diabetes-certified hospitals (DCH) vs. non-diabetes-certified hospitals (NDCH) from 2021 to 2023. Insights from this evaluation may support further refinement of inpatient diabetes care in Germany and offer context for similar accreditation initiatives internationally.

## 2 Materials and methods

### 2.1 Data source

Since 2004, all general hospitals in Germany have been required to submit data on their inpatient cases to the Institut für das Entgeltsystem im Krankenhaus (InEK) for accounting purposes. The nationwide diagnosis-related groups (DRG) statistics are official secondary statistics that comprise a legally defined selection of variables from these data, which are transmitted by the InEK to the German Federal Statistical Office and Statistical Offices of the Federal States (16). For this analysis, we applied to access to the DRG statistics for the 3 years 2021, 2022, and 2023 and we analyzed via controlled remote data processing all inpatient cases aged ≥20 years with diabetes as a primary or secondary diagnosis based on ICD-10 codes. A diagnosis of diabetes included type 1 diabetes or T1D (E10), type 2 diabetes or T2D (E11), other specified diabetes mellitus including pancreatic diabetes (diabetes resulting from diseases of the exocrine pancreas) (E13), rare types of diabetes (E12 or E14), and gestational diabetes (O24).

Several steps were necessary to identify the DCH and NDCH in the DRG statistics. First, we selected all centers providing inpatient care in the list of hospitals certified by the DDG (*n* = 867 as of 22.11.2023), excluding outpatient center as well as inpatient rehabilitation centers, since they are not part of the DRG statistics. Then, we selected all hospitals that had received at any time during the period 2021–2023 at least one of the DDG certificates [“Diabetes Excellence Centre”, “Diabetes Centre”, “Clinic with diabetes in focus” or “Certified treatment facility for diabetes foot” (15)] (*n* = 425). Thus, we did not differentiate in the present analysis between different types of certificates. To identify these centers in the DRG statistics, we have completed the list by adding the identification number of each hospital (“IK-Nummer”, available from the Quality report of the hospitals: https://www.g-ba.de/themen/qualitaetssicherung/datenerhebung-zur-qualitaetssicherung/datenerhebung-qualitaetsbericht/). Different hospital names with the same code were identified as one single institution. We finally obtained a list of 311 DCH with different identification codes. These codes were used to categorize the 1,415 hospitals from the DRG statistics for 2021–2023 in DCH and NDCH. Only hospitals that coded at least one diabetes case in the period 2021–2023 were finally included in the analysis (*n* = 1,403 hospitals, of which 300 DCH and 1,103 NDCH).

### 2.2 Statistical analysis

The following characteristics were described for all inpatient cases in DCH and NDCH: age, sex, length of hospital stays (days), in-hospital mortality (documentation of “death” as the reason for discharge from hospital), obesity (ICD-code: E66), hospital-acquired and procedure-related complications (online Supplementary Table S1: list of ICD-codes), hypoglycemia (E10-14.6), diabetic ketoacidosis, DKA (E10-14.1), and acute metabolic disorders with multiple complications (E10-14.73). Comparison between DCH and NDCH were conducted using Wilcoxon's rank sum test for continuous variables and *χ*^2^-test for dichotomous variables. *P*-values were adjusted for multiple testing according to the Holm-Bonferroni method.

Using linear and logistic mixed-effects regression models, we compared the mortality rate and the length of the hospital stay for inpatient cases with diabetes between DCH and NDCH. At patient level, we adjusted the models for sex, age, as well as proportion of obesity, emergency as reason for the admission, hospital-acquired and procedure-related complications, hypoglycemia, DKA, and acute metabolic disorder with multiple complications (these indicators were used as proxy measures for the severity of the patient's medical condition). At hospital level, we adjusted the models for the number of inpatient cases in the hospital (as proxy for the size of the hospital) and included the hospital as a random intercept to account for the hierarchical structure and the variability between hospitals. For inpatient cases with hypoglycemia or DKA, the same models were used without the adjustment for hypoglycemia, DKA, and acute metabolic disorder with multiple complications.

All regression analyses were performed for all inpatient cases with diabetes, as well as separately for those with T1D or T2D, and stratified according to whether diabetes was the reason for the hospital admission (primary diagnosis) or not (diabetes as a secondary diagnosis). We also analyzed more specifically the hospital stays with diabetic ketoacidosis (DKA) or hypoglycemia. In addition, we identified and compared the thirty most common reasons for admissions in people with diabetes in DCH and NDCH (online Supplementary Table S2) and performed the comparisons for inpatient cases with diabetes between DCH and NDCH stratified by the ten most common reasons for admission (online Supplementary Table S3). Results are presented as adjusted mortality rate and adjusted number of hospital days, as well as odds ratios (OR) for mortality in DCH vs. NDCH, with respective 95%-confidence intervals. The two-sided level of significance was set at 0.01. All analyses were performed with SAS version 9.4 (build TS1M8; SAS Institute, Inc., Cary, NC) on a window server mainframe.

## 3 Results

Overall, the DRG statistics recorded 43.4 million inpatient cases with or without diabetes in the 3-year period 2021–2023 in Germany (on average 14.5 million per year). A primary or secondary diagnosis of any type of diabetes was coded in 18.5% of all inpatient cases (*n* = 8,055,686 inpatient cases with diabetes, including 2,045 cases with T1D and T2D as double secondary diagnoses). The proportion of inpatient cases with diabetes was significantly higher in the certified hospitals (DCH: 19.0% vs. NDCH: 18.3%, *p* < 0.001). On average, one DCH handled more inpatient cases than one NDCH (18,542 vs. 8,078 cases per hospital per year) and more than twice as many inpatient cases with diabetes than one NDCH (3,519 vs. 1,477 cases per hospital per year). Nevertheless, given that the number of NDCH (*n* = 1,103) is much higher than that of DCH (*n* = 300), most hospital stays with diabetes (60.7%) occurred in NDCH (i.e., 4,888,749 inpatient cases with any type of diabetes in NDCH and 3,166,937 in DCH during the 3 years).

### 3.1 Description of all inpatient cases with diabetes in DCH vs. NDCH

Adults with diabetes admitted in DCH were slightly younger than in NDCH (mean: 71 vs. 72 years old, *p* < 0.001) and more often male (56% vs. 55%, *p* < 0.001) (Table 1). They also had higher rates of hospital-acquired and procedure-related complications (17.2% vs. 16.7%, *p* < 0.001), hypoglycemia (3.2% vs. 2.1%, *p* < 0.001), DKA (0.5% vs. 0.4%, *p* < 0.001), and acute metabolic disorder with multiple complications (2.3% vs. 1.0%, *p* < 0.001). However, the unadjusted length of stay and in-hospital mortality were similar in DCH and NDCH (mean length of stay 5 vs. 5 days (*p* = 0.034), and in-hospital mortality 4.5% vs. 4.5% (*p* = 0.061, Table 1).

**Table 1:** Comparison of inpatient cases with diabetes as primary or secondary diagnosis in diabetes-certified hospitals (DCH) and non-diabetes-certified hospitals (NDCH).

Variables	DCH (*n* = 300)	NDCH (*n* = 1,103)	*p*-values^a^
Number of inpatient cases with diabetes (2021–2023)	3,166,937	4,888,749	
Male	55.5	54.7	<0.001
Age (year)	70.8/73 (63–82)	72.0/74 (64–82)	<0.001
Obesity	11.1	11.9	<0.001
Length of stay (days)	8.1/5 (3–10)	8.2/5 (3–10)	0.034
Hospital-acquired and procedure-related complications^b^	17.2	16.7	<0.001
Hypoglycemia	3.2	2.1	<0.001
Diabetic ketoacidosis	0.5	0.4	<0.001
Acute metabolic disorders with multiple complications	2.3	1.0	<0.001
In-hospital mortality	4.5	4.5	0.061

Numbers presented are counts, percentages or mean/median (lower—upper quartile).

a Comparisons with Wilcoxon test for continuous variables or chi-squared test for binary variables. *P*-values adjusted for multiple testing according to the Holm-Bonferroni method.

b See list in Supplementary Table S1.

The thirty most common reasons for admissions in people with diabetes in DCH and NDCH (online Supplementary Table S2) covered 47.2% of all admissions in DCH and 46.5% of all admissions in NDCH. In both DCH and NDCH, cardiovascular diseases [ICD group of the “disease of the cardiovascular system (I00-I99)”] were the most frequent reason for hospital admission in people with diabetes, representing 47% and 44% of the thirty most frequent admission reasons in DCH and NDCH, respectively, with heart failure given as the most frequent reason in both DCH (6.0%) and NDCH (6.4%).

### 3.2 Description of inpatient cases with diabetes as primary diagnosis in DCH vs. NDCH

Diabetes itself was rarely documented as the reason for admission: only 0.2% of all inpatient cases had T1D as primary diagnosis (*n* = 71,875) and 0.9% T2D (*n* = 374,273). Diabetes as a primary diagnosis represented 31.8% of all cases with T1D and 5.0% of all cases with T2D.

Among all inpatient cases with diabetes, T1D as a primary diagnosis was twice as often documented in DCH (1.3%) than in NDCH (0.6%). These patients in DCH were slightly older (47.9 vs. 47.6 years old, *p* = 0.002), less frequently male than in NDCH (55% vs. 59%, *p* < 0.001). They had more episodes of hypoglycemia (20.1% vs. 16.0%) and acute metabolic disorders with multiple complications (21.5% vs. 6.6%, both *p* < 0.001), but DKA was less common (17.8% vs. 30.7%, *p* < 0.001) (Table 2a).

**Table 2a:** Comparison of inpatient cases with type 1 diabetes as primary diagnosis in diabetes-certified hospitals (DCH) and non-diabetes-certified hospitals (NDCH).

Variables	DCH (*n* = 300)	NDCH (*n* = 1,103)	*p*-values^a^
Number of inpatient cases with type 1 diabetes as primary diagnosis (2021–2023)	42,697	29,178	
Male	55.3	58.6	<0.001
Age (year)	47.9/47 (32–61)	47.6/46 (31–62)	0.002
Obesity	9.6	4.2	<0.001
Length of stay (days)	8.4/8 (4–10)	7.3/5 (3–9)	<0.001
Hospital-acquired and procedure-related complications^b^	10.2	9.5	0.003
Hypoglycemia	20.1	16.0	<0.001
Diabetic ketoacidosis	17.8	30.7	<0.001
Acute metabolic disorder with multiple complications	21.5	6.6	<0.001
In-hospital mortality	0.6	1.1	<0.001

Numbers presented are counts, percentages or mean/median (lower—upper quartile).

a Comparisons with Wilcoxon test for continuous variables or chi-squared test for binary variables. *P*-values adjusted for multiple testing according to the Holm-Bonferroni method.

b See list in Supplementary Table S1.

Among all inpatient diabetes cases, T2D as a primary diagnosis was also more often documented in DCH (5.6%) than in NDCH (4.0%). These patients in DCH were younger (69.2 vs. 71.2 years old, *p* < 0.001) and more often male (65% vs. 63%, *p* < 0.001) than in NDCH. They had more hospital-acquired and procedure-related complications (21.0% vs. 16.6%), and acute metabolic disorders with multiple complications (10.5% vs. 3.9%; both *p* < 0.001) (Table 2b).

**Table 2b:** Comparison of inpatient cases with type 2 diabetes as primary diagnosis in diabetes-certified hospitals (DCH) and non-diabetes-certified hospitals (NDCH).

Variables	DCH (*n* = 300)	NDCH (*n* = 1,103)	*p*-values^a^
Number of inpatient cases with type 2 diabetes as primary diagnosis (2021–2023)	177,587	196,686	
Male	64.7	62.8	<0.001
Age (year)	69.2/71 (60–80)	71.2/73 (62–82)	<0.001
Obesity	18.9	12.1	<0.001
Length of stay (days)	10.9/8 (5–13)	9.9/7 (4–12)	<0.001
Hospital-acquired and procedure-related complications^b^	21.0	16.6	<0.001
Hypoglycemia	13.2	14.6	<0.001
Diabetic ketoacidosis	2.5	3.2	<0.001
Acute metabolic disorder with multiple complications	10.5	3.9	<0.001
In-hospital mortality	2.3	2.9	<0.001

Numbers presented are counts, percentages or mean/median (lower—upper quartile).

a Comparisons with Wilcoxon test for continuous variables or chi-squared test for binary variables. *P*-values adjusted for multiple testing according to the Holm-Bonferroni method.

b See list in Supplementary Table S1.

Inpatient cases with diabetes as primary diagnosis presented more frequently with obesity in DCH (T1D: 9.6% vs. 4.2%; T2D: 18.9% vs. 12.1%; both *p* < 0.001). They also had longer hospital stays, but lower in-hospital mortality than in NDCH (T1D: 0.6% vs. 1.1%, T2D: 2.3% vs. 2.9%; both *p* < 0.001) (Tables 2a, 2b).

### 3.3 Mortality rate and length of hospital stay in DCH vs. NDCH

After adjustment for demographics, for the severity of the patient's medical condition, and for the size of the hospital, the mortality rate for all inpatient cases with diabetes was significantly lower in DCH than in NDCH (3.09% [95%-CI: 3.06–3.11] vs. 3.16% [3.14–3.18], *p* < 0.001) and the hospital stay shorter (7.90 days [7.76–8.04] vs. 9.00 days [8.91–9.09], *p* < 0.001) (Tables 3a, 3b). The absolute risk difference in mortality in DCH vs. NDCH was—7/10,000 inpatient cases (Table 3a).

**Table 3a:** In-hospital mortality for inpatient cases with diabetes in diabetes-certified hospitals (DCH) and non-diabetes-certified hospitals (NDCH).

Diagnosis	Adjusted in-hospital mortality in DCH [95%-CI]	Adjusted in-hospital mortality in NDCH [95%-CI]	*p*-values	Absolute risk differences in DCH vs. NDCH (/10,000 inpatient cases)
All types of diabetes	3.09 [3.06–3.11]	3.16 [3.14–3.18]	<0.001	−7
Type 1 diabetes				
All cases	0.96 [0.91–1.02]	1.14 [1.08–1.20]	<0.001	−18
As primary diagnosis	0.23 [0.19–0.28]	0.40 [0.33–0.47]	<0.001	−17
As secondary diagnosis	1.43 [1.35–1.52]	1.48 [1.40–1.56]	0.354	−5
Type 2 diabetes				
All cases	3.35 [3.32–3.38]	3.42 [3.40–3.44]	<0.001	−7
As primary diagnosis	1.44 [1.38–1.50]	1.64 [1.59–1.70]	<0.001	−20
As secondary diagnosis	3.47 [3.44–3.50]	3.49 [3.46–3.51]	0.283	−2
Any type of diabetes				
With DKA^a^	3.27 [3.00–3.56]	3.69 [3.42–3.98]	0.009	−42
With hypoglycemia^a^	4.72 [4.57–4.88]	5.87 [5.71–6.04]	<0.001	−115

Adjusted in-hospital mortality (%) in DCH and in NDCH, derived from mixed-effects logistic regression adjusted for sex, age, obesity, number of inpatient cases in the hospital, emergency as reason for the admission, hospital-acquired and procedure-related complications, hypoglycemia, diabetic ketoacidosis, acute metabolic disorder with multiple complications, and hospital as a random intercept.

a Without adjustment for hypoglycemia, diabetic ketoacidosis, and acute metabolic disorder with multiple complications.

**Table 3b:** Length of hospital stay for inpatient cases with diabetes in diabetes-certified hospitals (DCH) and non-diabetes-certified hospitals (NDCH).

Diagnosis	Adjusted length of hospital stay in DCH [95%-CI]	Adjusted length of hospital stay in NDCH [95%-CI]	*p*-values
All types of diabetes	7.90 [7.76–8.04]	9.00 [8.91–9.09]	<0.001
Type 1 diabetes			
All cases	7.12 [6.92–7.32]	7.33 [7.17–7.49]	0.086
As primary diagnosis	7.61 [7.39–7.83]	7.14 [6.97–7.32]	0.001
As secondary diagnosis	6.82 [6.64–6.99]	7.34 [7.20–7.49]	<0.001
Type 2 diabetes			
All cases	7.99 [7.85–8.13]	9.10 [9.01–9.19]	<0.001
As primary diagnosis	10.42 [10.16–10.68]	9.82 [9.64–10.01]	<0.001
As secondary diagnosis	7.82 [7.67–7.96]	9.04 [8.95–9.13]	<0.001
Any type of diabetes			
With DKA^a^	10.89 [10.58–11.19]	10.75 [10.49–11.00]	0.483
With hypoglycemia^a^	9.03 [8.83–9.24]	9.19 [9.03–9.34]	0.238

The length of hospital stay is given as adjusted mean estimate (in days) from mixed-effects linear regression adjusted for sex, age, obesity, number of inpatient cases in the hospital, emergency as reason for the admission, hospital-acquired and procedure-related complications, hypoglycemia, diabetic ketoacidosis, acute metabolic disorder with multiple complications, and hospital as a random intercept.

a Without adjustment for hypoglycemia, diabetic ketoacidosis, and acute metabolic disorder with multiple complications.

**Figure 1:**
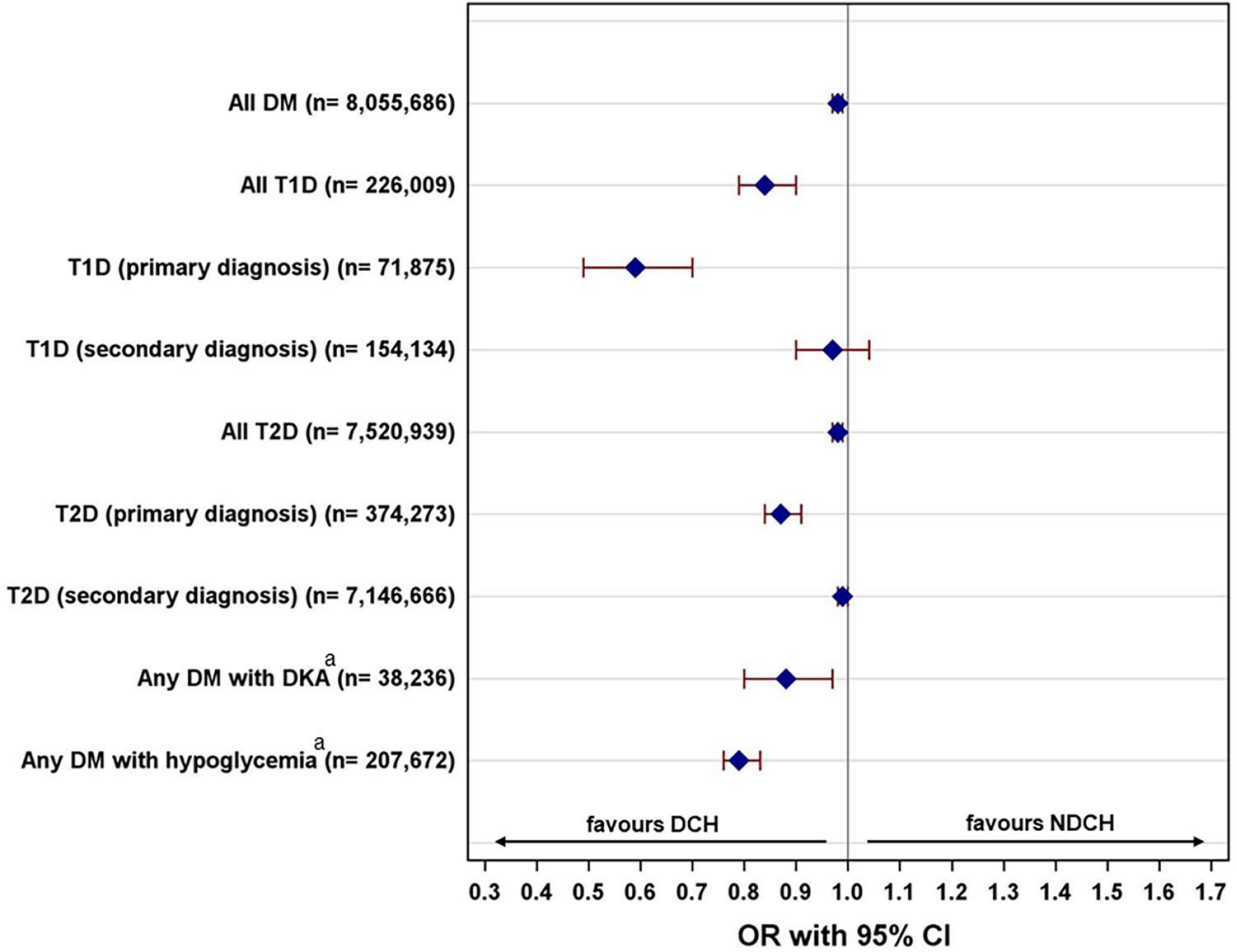
Adjusted odds ratios (OR) for in-hospital mortality for inpatient cases with diabetes in diabetes-certified hospitals (DCH) vs. non-diabetes-certified hospitals (NDCH). DM, diabetes mellitus; T1D, type 1 diabetes; T1D, type 1 diabetes; T2D, type 2 diabetes; DKA, diabetic ketoacidosis. Adjusted OR are derived from mixed-effects logistic regression, adjusted for sex, age, obesity, number of inpatient cases in the hospital, emergency as reason for the admission, hospital-acquired and procedure-related complications, hypoglycemia, diabetic ketoacidosis, acute metabolic disorder with multiple complications, and hospital as a random intercept. ^a^Without adjustment for hypoglycemia, diabetic ketoacidosis, and acute metabolic disorder with multiple complications.

Inpatient cases with diabetes as primary diagnosis had a significantly lower likelihood of mortality in DCH than in NDCH (OR for T1D: 0.59 [0.49–0.70], OR for T2D: 0.87 [0.84–0.91], all *p* < 0.001, Figure 1 and Supplementary Table S4), but a longer hospital stay (T1D: 7.61 days [7.39–7.83] vs. 7.14 days [6.97–7.32], *p* = 0.001, T2D: 10.42 days [10.16–10.68] vs. 9.82 days [9.64–10.01], *p* < 0.001, Table 3b).

Inpatient cases with diabetes as secondary diagnosis had a similar likelihood of mortality in DCH and in NDCH (OR for T1D: 0.97 [0.90–1.04], *p* = 0.354; OR for T2D: 0.99 [0.98–1.00], *p* = 0.283; Figure 1 and Supplementary Table S4), but a shorter hospital stay (T1D: 6.82 days [6.64–6.99] vs. 7.34 days [7.20–7.49], T2D: 7.82 days [7.67–7.96] vs. 9.04 days [8.95–9.13], all *p* < 0.001, Table 3b).

Patients admitted to hospital with hypoglycemia or DKA had a lower likelihood of mortality in DCH than in NDCH (OR with DKA: 0.88 [0.80–0.97], *p* = 0.009; OR with hypoglycemia: 0.79 [0.76–0.83], *p* < 0.001; Figure 1 and Supplementary Table S4), but a similar length of stay (Table 3b).

## 4 Discussion

This nationwide analysis of the mandatory DRG statistics, including more than 8 million inpatient cases with diabetes in the years 2021–2023, provides evidence, after multiple adjustments at both patient and hospital levels, that hospital stay in DCH is associated with a reduced mortality and a shorter hospital stay for patients with diabetes in Germany. The reduction in mortality was particularly evident among individuals admitted to hospital with T1D as primary diagnosis, who nearly halved their mortality when treated in certified facilities. In addition, adults with diabetes who were hospitalized for ketoacidosis or hypoglycemia had a 12% and 21% lower likelihood of in-hospital mortality, respectively, when treated in DCH.

Overall, the reduction in mortality in DCH vs. NDCH, with an absolute risk difference of—7/10,000 inpatient cases, is low. Nevertheless, taking into account the 4,888,749 inpatients cases with diabetes hospitalized in NDCH during the 3-years period, this result indicates that about 1,140 lives could have been saved each year. For people with T1D as main diagnosis, about 16 lives per year could have been saved, and around 131 for people with T2D as main diagnosis.

After stratification, we did not find a significant association between certification and in-hospital mortality when diabetes was coded as secondary diagnosis. In case of diabetes as secondary diagnosis, which is by far more frequent in T2D (95% of all cases with T2D compared to 68% for T1D), we can expect a high burden of comorbidities affecting mortality which cannot be influenced by adequate diabetes treatment. People with T2D admitted to the hospital were older than those with T1D. In addition to older age, T2D is frequently associated with other severe medical conditions beside T2D. Thus, it cannot be expected that mortality will decrease in the same extent as for diabetes as primary diagnosis or in cases with directly diabetes-related complications as a result of a greater focus of care on diabetes. However, for those mainly older and comorbid patients, we observed a significant shorter length of stay (about 1 day less) in DCH compared to NDCH.

Associations between treatment in certified centers and better outcomes, in particular lower mortality have been also found for other diseases in Germany. For example, several studies, which aimed to examine the effects of certification for oncology centers, reported better survival for patients with various types of cancer treated in hospitals certified by the German Cancer Society (17, 18). Improving the quality of inpatient diabetes care as well as patient safety in inpatient setting is a particularly important public health issue, as we have been able to confirm that nearly one in five inpatients in Germany carries a documented diagnosis of diabetes mellitus (4). Given the high number of unrecognized diabetes (1) and the potential underreporting of the secondary diagnosis of diabetes in the DRG statistics, the true prevalence of people with diabetes in hospitals is probably even higher (5). Our results indicate an association between diabetes-certified hospitals and reduced in-hospital mortality and shorter hospital stay, but reveal also that the majority of inpatients with diabetes in Germany, 61%, receive care in the NDCH.

DCH are overall larger hospitals than NDCH, handling more than twice as many inpatient cases with or without diabetes per hospital and per year than the NDCH. Larger hospitals are usually better equipped or have more personal resources, which are also criteria to obtain the certification. Therefore, more detailed studies, taking in account more precise hospital characteristics (size and structure of diabetology departments, existence of consultations services) are needed to investigate further in what extent the diabetes-specific expertise available in diabetes-certified hospitals can improve outcomes and help to manage diabetes more efficiently.

Prior research has shown that cardiovascular disease was the most common cause of hospital admission among adults in Germany in 2017, and that this primary diagnosis was significantly more frequent among people with diabetes in all age groups from 30 and above than among people without diabetes (4). It is well known that diabetes is associated with a 2 to 4-fold increased risk of cardiovascular disease, such as coronary heart disease, stroke, heart failure or peripheral artery disease (19). In line with these findings, we report that cardiovascular diseases remained the leading reason for admission among people with diabetes in 2021–2023, both in DCH and NDCH.

Notably, obesity appeared in only 11%–12% of all inpatient cases with diabetes, although 92.3% of them had T2D. The prevalence of obesity in people with T2D (45–79 years) in Germany is estimated at 54% (20). This underreporting of obesity in inpatient cases with diabetes in the DRG statistics may have several explanations. One of these is that the DRG statistics consists in secondary data which are primarily collected for billing purposes, and not for research. Therefore, clinical information may be lacking and comorbidities may be underestimated if the condition is not directly relevant for billing. It is also likely that mainly cases of extreme obesity are coded (21). We observed that obesity was coded more frequently in people with a primary diagnosis of diabetes, especially in DCH. It is possible that in DCH, more adults with diabetes and severe obesity are treated. Another possibility is that secondary diagnoses of obesity are better documented in DCH, especially when diabetes is the reason for admission.

### 4.1 Strengths and limitations

A major strength of this study is the use of mandatory nationwide DRG data, which captures almost all acute-care hospital admissions in Germany (except for psychiatric and psychosomatic hospitals and rehabilitation facilities). Including both primary and secondary diagnoses of diabetes, this study describes the real prevalence of documented diabetes in hospitals.

The descriptive part of our study revealed that patients in DCH generally presented with more complications, hypoglycemia, and acute metabolic disorders, suggesting a higher severity of illness. Patients with diabetes as the primary diagnosis more often presented with obesity, complications, and acute metabolic disorders with multiple complications in DCH. We therefore adjusted all regression models for several potential confounders, including the severity of the inpatient cases and the size of the hospitals, to enable comparisons of inpatient cases between the DCH and in the NDCH while reducing the risk of biases. However, residual confounding—related, for example, to socioeconomic and ethnic differences at individual level or to regional and socioeconomic variation between hospitals—may still remain.

Some limitations are inherent to the nature of the DRG statistics that are secondary data not primarily collected for research, but for billing reasons. In the present analysis, we used all cases with diabetes as “primary diagnosis” (reason for admission) or as secondary diagnosis (medical conditions or comorbidities not declared as the leading cause for hospital admission) as these categories are predefined in the mandatory DRG-system. However, we do not expect substantial differences in coding between DCH and NCDH. Every hospital has coding specialists trained to code diagnoses of inpatient cases in accordance with strict legal requirements. In addition, coding is subject to review by the Medical Service of the Health Insurance Funds. Nevertheless, the financial aspects of coding must not be overlooked. As a primary diagnosis of diabetes generates less revenue for the hospital than, for example, a primary diagnosis of kidney failure, it is likely that the “primary diagnosis of diabetes” is underrepresented as a reason for hospital admission.

Another limitation is that the identification of the DCH by the institution code of the hospitals does not make it possible to differentiate between the different types of DDG certificates, as different departments of a hospital can have different certificates under a single institution code. It is also not possible to draw conclusions about which specialist departments within a hospital are actually certified for diabetes based on the institution code. The vast majority of people with diabetes who were treated as inpatients were admitted for other primary diagnoses, and therefore even in the DCH, many cases might not have been treated in the actual certified diabetes departments of the hospitals, but in other departments. However, it should be assumed that people with diabetes admitted to DCH as inpatients were in most departments also treated for their diabetes by a diabetology-certified consultation service (“diabetes unit”).

Finally, a limitation is that the DRG statistics relate to inpatient “cases” (hospital admissions) and are subject to strict data protection rules, which makes it impossible to draw conclusions at the patient level or regarding the actual number of patients.

## 5 Conclusions

This nationwide analysis provides first evidence that hospitalized people with diabetes in diabetes certified hospitals have reduced odds of in-hospital mortality, when the reason for hospitalization is diabetes itself or when they are admitted to hospital with directly diabetes-related complications such as ketoacidosis or hypoglycemia. This analysis also indicates that people with diabetes admitted to certified hospitals for other reasons do not have a significantly reduced in-hospital mortality, but experience in mean a 1-day shorter hospital stay. More detailed analyses are needed to confirm and deepen these results. In particular, prospective comparisons, stratified by hospital characteristics, would be helpful to clarify in which extent the diabetes-specific expertise available in diabetes-certified hospitals contribute to improve outcomes of hospitalized people with diabetes and to manage diabetes more efficiently.

## Data Availability

Publicly available datasets were analyzed in this study. This data can be found here: Research data centre (RDC) of the Federal Statistical Office and Statistical Offices of the Federal States, DOI: 10.21242/23141.2021.00.00.6.1.0; 10.21242/23141.2022.00.00.6.1.0; 10.21242/23141.2023.00.00.6.1.0.
