# Supplementary material for: In-hospital outcomes of adults with diabetes treated in certified vs. non-certified hospitals: a nationwide analysis of German DRG statistics
Source: Front Health Serv. 2026 May 21;6:1782882. doi: 10.3389/frhs.2026.1782882 (PMC13233407; doi:10.3389/frhs.2026.1782882)
Supplement: Supplementary file 1 [file Table1.docx]

**Supplemental table 1. ICD-10 codes and diagnoses that were combined as “Hospital-acquired and procedure-related complications”**

| **ICD-10 Code** | **Diagnoses** |
| --- | --- |
| **E89.0** | Postprocedural hypothyroidism |
| **E89.1** | Postprocedural hypoinsulinaemia |
| **E89.2** | Postprocedural hypoparathyroidism |
| **E89.3** | Postprocedural hypopituitarism |
| **E89.4** | Postprocedural ovarian failure |
| **E89.5** | Postprocedural testicular hypofunction |
| **E89.6** | Postprocedural adrenocortical(-medullary) hypofunction |
| **E89.8** | Other postprocedural endocrine and metabolic disorders |
| **E89.9** | Postprocedural endocrine and metabolic disorder, unspecified |
| **G97.0** | Cerebrospinal fluid leak from spinal puncture |
| **G97.1** | Other reaction to spinal and lumbar puncture |
| **G97.2** | Intracranial hypotension following ventricular shunting |
| **G97.8** | Other postprocedural disorders of nervous system |
| **G97.9** | Postprocedural disorder of nervous system, unspecified |
| **H59.0** | Keratopathy (bullous aphakic) following cataract surgery |
| **H59.8** | Other postprocedural disorders of eye and adnexa |
| **H59.9** | Postprocedural disorder of eye and adnexa, unspecified |
| **H95.0** | Recurrent cholesteatoma of postmastoidectomy cavity |
| **H95.1** | Other disorders following mastoidectomy |
| **H95.8** | Other postprocedural disorders of ear and mastoid process |
| **H95.9** | Postprocedural disorder of ear and mastoid process, unspecified |
| **I97.0** | Postcardiotomy syndrome |
| **I97.1** | Other functional disturbances following cardiac surgery |
| **I97.2** | Postmastectomy lymphoedema syndrome |
| **I97.8** | Other postprocedural disorders of circulatory system, not elsewhere classified |
| **I97.9** | Postprocedural disorder of circulatory system, unspecified |
| **J95.0** | Tracheostomy malfunction |
| **J95.1** | Acute pulmonary insufficiency following thoracic surgery |
| **J95.2** | Acute pulmonary insufficiency following nonthoracic surgery |
| **J95.3** | Chronic pulmonary insufficiency following surgery |
| **J95.4** | Mendelson syndrome |
| **J95.5** | Postprocedural subglottic stenosis |
| **J95.8** | Other postprocedural respiratory disorders |
| **J95.9** | Postprocedural respiratory disorder, unspecified |
| **K91.0** | Vomiting following gastrointestinal surgery |
| **K91.1** | Postgastric surgery syndromes |
| **K91.2** | Postsurgical malabsorption, not elsewhere classified |
| **K91.3** | Postoperative intestinal obstruction |
| **K91.4** | Colostomy and enterostomy malfunction |
| **K91.5** | Postcholecystectomy syndrome |
| **K91.8** | Other postprocedural disorders of digestive system, not elsewhere classified |
| **K91.9** | Postprocedural disorder of digestive system, unspecified |
| **L89.0** | Stage I decubitus ulcer and pressure area |
| **L89.1** | Stage II decubitus ulcer |
| **L89.2** | Stage III decubitus ulcer |
| **L89.3** | Stage IV decubitus ulcer |
| **L89.9** | Decubitus ulcer and pressure area, unspecified |
| **M96.0** | Pseudarthrosis after fusion or arthrodesis |
| **M96.1** | Postlaminectomy syndrome, not elsewhere classified |
| **M96.2** | Postradiation kyphosis |
| **M96.3** | Postlaminectomy kyphosis |
| **M96.4** | Postsurgical lordosis |
| **M96.5** | Postradiation scoliosis |
| **M96.6** | Fracture of bone following insertion of orthopaedic implant, joint prosthesis, or bone plate |
| **M96.8** | Other postprocedural musculoskeletal disorders |
| **M96.9** | Postprocedural musculoskeletal disorder, unspecified |
| **N99.0** | Postprocedural renal failure |
| **N99.1** | Postprocedural urethral stricture |
| **N99.2** | Postoperative adhesions of vagina |
| **N99.3** | Prolapse of vaginal vault after hysterectomy |
| **N99.4** | Postprocedural pelvic peritoneal adhesions |
| **N99.5** | Malfunction of external stoma of urinary tract |
| **N99.8** | Other postprocedural disorders of genitourinary system |
| **N99.9** | Postprocedural disorder of genitourinary system, unspecified |
| **T80.0** | Air embolism following infusion, transfusion and therapeutic injection |
| **T80.1** | Vascular complications following infusion, transfusion and therapeutic injection |
| **T80.2** | Infections following infusion, transfusion and therapeutic injection |
| **T80.3** | ABO incompatibility reaction |
| **T80.4** | Rh incompatibility reaction |
| **T80.5** | Anaphylactic shock due to serum |
| **T80.6** | Other serum reactions |
| **T80.8** | Other complications following infusion, transfusion and therapeutic injection |
| **T80.9** | Unspecified complication following infusion, transfusion and therapeutic injection |
| **T81.0** | Haemorrhage and haematoma complicating a procedure, not elsewhere classified |
| **T81.1** | Shock during or resulting from a procedure, not elsewhere classified |
| **T81.2** | Accidental puncture and laceration during a procedure, not elsewhere classified |
| **T81.3** | Disruption of operation wound, not elsewhere classified |
| **T81.4** | Infection following a procedure, not elsewhere classified |
| **T81.5** | Foreign body accidentally left in body cavity or operation wound following a procedure |
| **T81.6** | Acute reaction to foreign substance accidentally left during a procedure |
| **T81.7** | Vascular complications following a procedure, not elsewhere classified |
| **T81.8** | Other complications of procedures, not elsewhere classified |
| **T81.9** | Unspecified complication of procedure |
| **T82.0** | Mechanical complication of heart valve prosthesis |
| **T82.1** | Mechanical complication of cardiac electronic device |
| **T82.2** | Mechanical complication of coronary artery bypass and valve grafts |
| **T82.3** | Mechanical complication of other vascular grafts |
| **T82.4** | Mechanical complication of vascular dialysis catheter |
| **T82.5** | Mechanical complication of other cardiac and vascular devices and implants |
| **T82.6** | Infection and inflammatory reaction due to cardiac valve prosthesis |
| **T82.7** | Infection and inflammatory reaction due to other cardiac and vascular devices, implants and grafts |
| **T82.8** | Other specified complications of cardiac and vascular prosthetic devices, implants and grafts |
| **T82.9** | Unspecified complication of cardiac and vascular prosthetic device, implant and graft |
| **T83.0** | Mechanical complication of urinary (indwelling) catheter |
| **T83.1** | Mechanical complication of other urinary devices and implants |
| **T83.2** | Mechanical complication of graft of urinary organ |
| **T83.3** | Mechanical complication of intrauterine contraceptive device |
| **T83.4** | Mechanical complication of other prosthetic devices, implants and grafts in genital tract |
| **T83.5** | Infection and inflammatory reaction due to prosthetic device, implant and graft in urinary system |
| **T83.6** | Infection and inflammatory reaction due to prosthetic device, implant and graft in genital tract |
| **T83.8** | Other complications of genitourinary prosthetic devices, implants and grafts |
| **T83.9** | Unspecified complication of genitourinary prosthetic device, implant and graft |
| **T84.0** | Mechanical complication of internal joint prosthesis |
| **T84.1** | Mechanical complication of internal fixation device of bones of limb |
| **T84.2** | Mechanical complication of internal fixation device of other bones |
| **T84.3** | Mechanical complication of other bone devices, implants and grafts |
| **T84.4** | Mechanical complication of other internal orthopaedic devices, implants and grafts |
| **T84.5** | Infection and inflammatory reaction due to internal joint prosthesis |
| **T84.6** | Infection and inflammatory reaction due to internal fixation device [any site] |
| **T84.7** | Infection and inflammatory reaction due to other internal orthopaedic prosthetic devices, implants and grafts |
| **T84.8** | Other complications of internal orthopaedic prosthetic devices, implants and grafts |
| **T84.9** | Unspecified complication of internal orthopaedic prosthetic device, implant and graft |
| **T85.0** | Mechanical complication of ventricular intracranial (communicating) shunt |
| **T85.1** | Mechanical complication of implanted electronic stimulator of nervous system |
| **T85.2** | Mechanical complication of intraocular lens |
| **T85.3** | Mechanical complication of other ocular prosthetic devices, implants and grafts |
| **T85.4** | Mechanical complication of breast prosthesis and implant |
| **T85.5** | Mechanical complication of gastrointestinal prosthetic devices, implants and grafts |
| **T85.6** | Mechanical complication of other specified internal prosthetic devices, implants and grafts |
| **T85.7** | Infection and inflammatory reaction due to other internal prosthetic devices, implants and grafts |
| **T85.8** | Other complications of internal prosthetic devices, implants and grafts, not elsewhere classified |
| **T85.9** | Unspecified complication of internal prosthetic device, implant and graft |
| **T86.0** | Bone-marrow transplant rejection |
| **T86.1** | Kidney transplant failure and rejection |
| **T86.2** | Heart transplant failure and rejection |
| **T86.3** | Heart-lung transplant failure and rejection |
| **T86.4** | Liver transplant failure and rejection |
| **T86.8** | Failure and rejection of other transplanted organs and tissues |
| **T86.9** | Failure and rejection of unspecified transplanted organ and tissue |
| **T87.0** | Complications of reattached (part of) upper extremity |
| **T87.1** | Complications of reattached (part of) lower extremity |
| **T87.2** | Complications of other reattached body part |
| **T87.3** | Neuroma of amputation stump |
| **T87.4** | Infection of amputation stump |
| **T87.5** | Necrosis of amputation stump |
| **T87.6** | Other and unspecified complications of amputation stump |
| **T88.0** | Infection following immunization |
| **T88.1** | Other complications following immunization, not elsewhere classified |
| **T88.2** | Shock due to anaesthesia |
| **T88.3** | Malignant hyperthermia due to anaesthesia |
| **T88.4** | Failed or difficult intubation |
| **T88.5** | Other complications of anaesthesia |
| **T88.6** | Anaphylactic shock due to adverse effect of correct drug or medicament properly administered |
| **T88.7** | Unspecified adverse effect of drug or medicament |
| **T88.8** | Other specified complications of surgical and medical care, not elsewhere classified |
| **T88.9** | Complication of surgical and medical care, unspecified |
| **U69.0** | Elsewhere classified hospital-acquired pneumonia |

**Supplemental table 2.** Thirty most frequent causes of hospital admission (primary diagnoses) for inpatient cases with diabetes in diabetes-certified hospitals (DCH) and non-diabetes-certified hospitals (NDCH)

|  | **DCH** | **(%)** | **NDCH** | **(%)** |
| --- | --- | --- | --- | --- |
| **1** | Heart failure (I50) | 6.0 | Heart failure (I50) | 6.4 |
| **2** | Type 2 diabetes mellitus (E11) | 5.6 | Type 2 diabetes mellitus (E11) | 4.0 |
| **3** | Arteriolosclerosis (I70) | 2.7 | Cerebral infarction (I63) | 2.7 |
| **4** | Cerebral infarction (I63) | 2.4 | Arteriolosclerosis (I70) | 2.1 |
| **5** | Atrial fibrillation and flutter (I48) | 2.3 | Atrial fibrillation and flutter (I48) | 2.1 |
| **6** | Chronic ischemic heart disease (I25) | 2.3 | Acute myocardial infarction (I21) | 2.0 |
| **7** | Acute myocardial infarction (I21) | 2.2 | Chronic ischemic heart disease (I25) | 1.8 |
| **8** | Angina pectoris (I20) | 2.0 | Other disorders of urinary system (N39) | 1.8 |
| **9** | Other disorders of urinary system (N39) | 1.6 | Fracture of femur (S72) | 1.7 |
| **10** | Other chronic obstructive pulmonary disease (J44) | 1.4 | Other chronic obstructive pulmonary disease (J44) | 1.7 |
| **11** | Fracture of femur (S72) | 1.4 | Angina pectoris (I20) | 1.6 |
| **12** | Type 1 diabetes mellitus (E10) | 1.3 | Pneumonia (J18) | 1.4 |
| **13** | Viral pneumonia (J20) | 1.3 | Viral pneumonia (J12) | 1.3 |
| **14** | Pneumonia (J18) | 1.2 | Volume depletion (E86) | 1.3 |
| **15** | Malignant neoplasm of bronchus and lung (C34) | 1.2 | Osteoarthritis of knee (M17) | 1.3 |
| **16** | Cholelithiasis (K80) | 1.1 | Malignant neoplasm of bronchus and lung (C34) | 1.2 |
| **17** | Volume depletion (E86) | 1.0 | Cholelithiasis (K80) | 1.1 |
| **18** | Acute kidney failure (N17) | 1.0 | Essential (primary) hypertension (I10) | 1.1 |
| **19** | Essential (primary) hypertension (I10) | 0.9 | Intracranial injury (S06) | 1.0 |
| **20** | Intracranial injury (S06) | 0.9 | Osteoarthritis of hip (M16) | 1.0 |
| **21** | Obstructive and reflux uropathy (N13) | 0.8 | Acute kidney failure (N17) | 0.9 |
| **22** | Malignant neoplasm of pancreas (C25) | 0.8 | Dorsalgia (M54) | 0.9 |

| **23** | Malignant neoplasm of bladder (C67) | 0.8 | Other spondylopathies (M48) | 0.8 |
| --- | --- | --- | --- | --- |
| **24** | Transient cerebral ischemic attacks and related syndromes (G45) | 0.8 | Obstructive and reflux uropathy (N13) | 0.8 |
| **25** | Nonrheumatic aortic valve disorders (I35) | 0.8 | Transient cerebral ischemic attacks and related syndromes (G45) | 0.8 |
| **26** | Other sepsis (A41) | 0.7 | Other sepsis (A41) | 0.8 |
| **27** | Osteoarthritis of knee (M17) | 0.7 | Malignant neoplasm of bladder (C67) | 0.7 |
| **28** | T82 Complications of cardiac and vascular prosthetic devices, implants and grafts | 0.7 | Gastritis and duodenitis (K29) | 0.7 |
| **29** | C44 Other and unspecified malignant neoplasm of skin | 0.7 | Nonrheumatic aortic valve disorders (I35) | 0.7 |
| **30** | Syncope and collapse (R55) | 0.6 | Fracture of lumbar spine and pelvis (S32) | 0.7 |

**Supplemental table 3a.** In-hospital mortality in diabetes-certified hospitals (DCH) and non-diabetes-certified hospitals (NDCH), for inpatients cases with diabetes, stratified by the ten most frequent causes of hospital admission for adults with diabetes†

| **Cause of hospital admission** | **DCH**  **(n= 300)** | **NDCH**  **(n= 1,103)** | **Adjusted OR**  **[95%-CI]** | **p-values** |
| --- | --- | --- | --- | --- |
| Heart failure | 7.51 [7.36-7.66] | 7.51 [7.39-7.62] | 1.00 [0.97-1.03] | 0.984 |
| Atherosclerosis | 1.89 [1.80-1.99] | 1.99 [1.90-2.08] | 0.95 [0.90-1.00] | 0.098 |
| Cerebral infarction | 5.86 [5.65-6.07] | 5.71 [5.55-5.86] | 1.03 [0.98-1.08] | 0.241 |
| Atrial flutter and atrial fibrillation | 0.48 [0.44-0.53] | 0.48 [0.44-0.53] | 1.01 [0.91-1.12] | 0.871 |
| Chronic ischemic heart disease | 0.56 [0.51-0.62] | 0.59 [0.54-0.64] | 0.96 [0.86-1.06] | 0.398 |
| Myocardial infarction | 6.99 [6.81-7.18] | 6.98 [6.76-7.20] | 1.00 [0.96-1.04] | 0.912 |
| Angina pectoris | 2.42 [2.08-2.81] | 2.32 [2.01-2.67] | 1.04 [0.88-1.24] | 0.630 |
| Other disorders of urinary system | 3.26 [3.11-3.43] | 3.11 [2.99-3.23] | 1.05 [0.99-1.12] | 0.105 |
| Fracture of femur | 5.48 [5.25-5.71] | 5.41 [5.25-5.59] | 1.01 [0.96-1.07] | 0.646 |
| COPD | 4.68 [4.47-4.88] | 4.66 [4.50-4.82] | 1.00 [0.95-1.06] | 0.880 |

Adjusted In-hospital mortality and odds-ratios (OR) for comparison between DCH and NDCH are derived from mixed-effects logistic regression adjusted for sex, age, obesity, number of inpatient cases in the hospital, emergency as reason for the admission, hospital-acquired and procedure-related complications, and hospital as a random intercept.

† See online supplemental table 2.

**Supplemental table 3b.** Length of hospital stay in diabetes-certified hospitals (DCH) and non-diabetes-certified hospitals (NDCH). for inpatients cases with diabetes, stratified by the ten most frequent causes of hospital admission for adults with diabetes†

| **Cause of hospital admission** | **Adjusted in-hospital mortality**  **in DCH [95%-CI]** | **Adjusted in-hospital mortality**  **in NDCH [95%-CI]** | **p-values** |
| --- | --- | --- | --- |
| Heart failure | 10.71 [10.51-10.92] | 10.23 [10.08-10.38] | <0.001* |
| Atherosclerosis | 10.30 [9.95-10.64] | 10.56 [10.28-10.83] | 0.247 |
| Cerebral infarction | 10.88 [10.24-11.52] | 12.92 [12.44-13.39] | 0.022 |
| Atrial flutter and atrial fibrillation | 4.62 [4.50-4.74] | 4.61 [4.51-4.70] | 0.876 |
| Chronic ischemic heart disease | 4.30 [4.00-4.60] | 4.79 [4.55-5.03] | 0.010 |
| Myocardial infarction | 8.01 [7.81-8.21] | 7.80 [7.63-7.96] | 0.093 |
| Angina pectoris | 3.84 [3.66-4.01] | 3.76 [3.61-3.90] | 0.484 |
| Other disorders of urinary system | 7.93 [7.78-8.09] | 7.86 [7.75-7.98] | 0.459 |
| Fracture of femur | 15.21 [14.84-15.57] | 14.71 [14.46-14.97] | 0.032 |
| COPD | 8.67 [8.48-8.86] | 8.45 [8.31-8.59] | 0.069 |

The length of hospital stay is given as adjusted mean estimate (in days) from mixed-effects linear regression adjusted for sex, age, obesity, number of inpatient cases in the hospital, emergency as reason for the admission, hospital-acquired and procedure-related complications, and hospital as a random intercept.

† See online supplemental table 2.

**Supplemental table 4.** Odds-ratios for in-hospital mortality for inpatient cases with diabetes in diabetes-certified hospitals (DCH) vs. non-diabetes-certified hospitals (NDCH)

|  | **Adjusted OR [95%-CI]**  **for in-hospital mortality in DCH vs. NDCH** | **p-values** |
| --- | --- | --- |
| **All types of diabetes** | 0.98 [0.97-0.99] | <0.001* |
| **Type 1 diabetes** |  |  |
| All cases | 0.84 [0.79-0.90] | <0.001* |
| as primary diagnosis | 0.59 [0.49-0.70] | <0.001* |
| as secondary diagnosis | 0.97 [0.90-1.04] | 0.354 |
| **Type 2 diabetes** |  |  |
| All cases | 0.98 [0.97-0.99] | <0.001* |
| as primary diagnosis | 0.87 [0.84-0.91] | <0.001* |
| as secondary diagnosis | 0.99 [0.98-1.00] | 0.283 |
| **Any type of diabetes** |  |  |
| with DKA† | 0.88 [0.80-0.97] | 0.009* |
| with hypoglycemia† | 0.79 [0.76-0.83] | < 0.001* |

Odds-ratios (OR) for in-hospital mortality for inpatient cases with diabetes in diabetes-certified hospitals (DCH) vs. non-diabetes-certified hospitals (NDCH), derived from mixed-effects logistic regression adjusted for sex, age, obesity, number of inpatient cases in the hospital, emergency as reason for the admission, hospital-acquired and procedure-related complications, hypoglycemia, diabetic ketoacidosis, acute metabolic disorder with multiple complications, and hospital as a random intercept.

† Without adjustment for hypoglycemia, diabetic ketoacidosis, and acute metabolic disorder with multiple complications.
